# Clinicopathological features and outcome of chronic lymphocytic leukaemia in Chinese patients

**DOI:** 10.18632/oncotarget.16037

**Published:** 2017-03-09

**Authors:** Thomas Sau-Yan Chan, Yuh-Shan Lee, Ilaria Del Giudice, Marilisa Marinelli, Caterina Ilari, Luciana Cafforio, Anna Guarini, Daryl Tan, Colin Phipps, Yeow-Tee Goh, William Hwang, Allan Zhi-Kai Goh, Lisa Lai-Ping Siu, Saliangi Wu, Chun-Yin Ha, Shek-Ying Lin, Chi-Hang Kwok, Chi-Kuen Lau, Kit-Fai Wong, Robin Foà, Yok-Lam Kwong, Eric Tse

**Affiliations:** ^1^ Department of Medicine, The University of Hong Kong, Queen Mary Hospital, Hong Kong, China; ^2^ Department of Haematology, Singapore General Hospital, Outram, Singapore; ^3^ Department of Cellular Biotechnologies and Hematology, Sapienza University, Rome, Italy; ^4^ Department of Molecular Medicine, Sapienza University, Rome, Italy; ^5^ Department of Pathology, Queen Elizabeth Hospital, Hong Kong, China; ^6^ Department of Medicine, Queen Elizabeth Hospital, Hong Kong, China; ^7^ Department of Medicine, Tuen Mun Hospital, Hong Kong, China; ^8^ Department of Medicine, United Christian Hospital, Hong Kong, China; ^9^ Department of Medicine, Princess Margaret Hospital, Hong Kong, China; ^10^ Department of Medicine, Tseung Kwan O Hospital, Hong Kong, China

**Keywords:** chronic lymphocytic leukaemia, Chinese, pathological characteristics, clinical outcomes, prognostication

## Abstract

Chronic lymphocytic leukaemia (CLL) is uncommon in Chinese population and its biology, genetics and treatment outcome in Chinese patients have not been comprehensively investigated. In this study, we studied the clinicopathological features and outcome of 212 Chinese patients with newly diagnosed CLL in Hong Kong and Singapore. The median age at diagnosis was 64 years. The majority of patients presented with early-stage disease (Binet stage A, 56.1%). Del(13)(q14) was the most frequent abnormality (41.7%) detected by fluorescence *in situ* hybridization (FISH) analysis. Del(17p) and *TP53* gene mutations were detected in 7.8% and 8.2% of patients, respectively. *MYD88* mutations were found at a higher frequency (11.5%) than expected. CLL with unmutated variable region of the immunoglobulin heavy chain genes (*IGHV*) occurred in only 31.2% of cases, and was associated with advanced-stage disease (p <0.01) and adverse FISH abnormalities (p<0.01). With a median follow-up of 39 months, the median overall survival (OS) was 108 months. The presence of del(17p) or *TP53* mutations was associated with a significantly shorter time to first treatment and an inferior OS (p <0.01). Unmutated *IGHV* was also associated with a significantly shorter time to treatment (p <0.01). Among patients who required treatment, the median OS and progression-free survival (PFS) were 107 and 23 months, respectively. The presence of del(17p) was associated with a significantly inferior OS and PFS (p <0.01). In summary, Chinese CLL patients had similar genetic aberrations at diagnosis compared with those of Western populations. FISH abnormalities are major factors affecting outcome.

## INTRODUCTION

Chronic lymphocytic leukaemia (CLL) is a mature B-cell neoplasm. It is characterized by an increased number of circulating clonal B-cells (>5 × 10^9^/L) with typical morphology and immunophenotype (CD5^+^, CD23^+^, CD79b^weak/−^, FMC7^weak/−^, surface immunoglobulin^dim^) [1]. It is the most common leukaemia in the Western world, accounting for 25% of all leukaemias in adults. The age-adjusted annual incidence rate (AAIR) of CLL in USA is 4.75 per 100,000 persons [2]. In contrast, CLL is much rarer in Asian populations, with an AAIR of less than 0.5 per 100,000 persons [3, 4]. Epidemiological studies also revealed a similarly low incidence of CLL in Asian migrants in North America, underscoring the importance of genetics in the pathogenesis of CLL [4, 5]. Nonetheless, the genetic basis for such ethnical differences remains undefined [6].

CLL is clinically heterogeneous. In some patients, the disease remains indolent and asymptomatic for years. Other patients, however, experience rapid disease progression even after aggressive treatment. The clinical staging systems devised by Rai et al [7] and Binet et al [8] are widely adopted for risk stratification and as an indicator for treatment in the International Workshop on CLL (iwCLL) guidelines [9]. More recently, recurrent cytogenetic and molecular aberrations identified in CLL have provided important information on the genomic landscape of the disease. With karyotyping and fluorescence *in situ* hybridization (FISH), a hierarchal prognostic model has been proposed [10]. Deletion of the short arm of chromosome 17, del(17p), is associated with the worst prognosis, which is not ameliorated by conventional chemoimmunotherapy [11]. The mutation status of the immunoglobulin heavy chain variable region (*IGHV*) genes is also a powerful predictor of outcome, with mutated *IGHV* being associated with superior survival [12, 13]. The use of conventional and/or next generation sequencing has also identified genetic mutations in the *TP53*, *NOTCH1*, *BIRC3* and *SF3B1* genes, which are of prognostic significance [14–21].

Currently, existing information on cytogenetic and molecular studies has been derived from Caucasian populations, and available data for CLL in Chinese patients are very limited. Comparing the cytogenetic and molecular features of CLL in Chinese and Caucasian patients may help to explain the marked ethnical disparities in disease incidence. Furthermore, it is also necessary to define the clinical significance and impact of prognostic or predictive biomarkers derived from Western patients when applied to Asian populations. To address these issues, we studied the clinical, pathological and molecular characteristics of CLL in a cohort of Chinese patients from Hong Kong and Singapore.

## RESULTS

### Demographical and clinical characteristics

Two hundred and twelve patients (men, N=154; and women, N=58) at a median age of 64 (26 – 94) years were studied (Hong Kong cohort, retrospective group, N=44, prospective group, N=95; Singapore cohort, N=73). More than half of the patients presented with early-stage disease (Binet stage A, 56.1%). The median white blood cell count and lymphocyte count at the time of diagnosis were 28 x10^9^/L (6.2 - 453 x10^9^/L) and 20.8 × 10^9^/L (5.2 - 448.3 x10^9^/L). The demographical and baseline clinical characteristics of the patients were summarized in Table 1.

**Table 1:** Demographical and clinical characteristics at baseline

**Characteristics**	
Gender	
Male	N = 154 (73%)
Female	N = 58 (27%)
Median age (range)	64 years (26-94 years)
Blood counts on presentation	
Total white blood cells	28 × 10^9^/L (6.2 - 453 × 10^9^/L)
Lymphocytes	20.76 × 10^9^/L (5.24 – 448.3 × 10^9^/L)
Haemoglobin	12.5 g/dL (4.5 – 18 g/dL)
Platelets	182 × 10^9^/L (22-895 × 10^9^/L)
Binet stage	
A	N = 119 (56.1%)
B	N = 46 (21.7%)
C	N = 47 (22.2%)
Rai stage	
0	N = 59 (27.8%)
I	N = 55 (26.0%)
II	N= 45 (21.2%)
III	N= 28 (13.2%)
IV	N= 25 (11.8%)

### FISH analysis and conventional karyotyping

FISH analysis was performed in 192 cases (Table 2). The most frequent abnormality was del(130(q14) (N=80, 41.7%), followed by trisomy 12 (N=28, 14.6%), del(11)(q23) (N=22, 11.4%) and del(17p) (N=15, 7.8%). Forty-seven (24.5%) patients showed no abnormality with the FISH probes used. Conventional karyotyping was performed in 139 patients. Complex cytogenetic changes (defined as ≥3 chromosomal abnormalities) were found in 12 (8.6%) patients (Table 2). FISH analysis was available in 11 of these cases, and 4 of them (36%) showed del(17p).

**Table 2:** Results of FISH, karyotyping, *IGHV* analysis and targeted gene sequencing

FISH abnormalities	*N* (%)^#^Current study	N (%)Dohner et al [10]	N (%)Van Dyke et al[28]
Total	192 (100%)	325 (100%)	1048 (100%)
Deletion 13q14 (as sole abnormality)	80 (41.7%)	117 (36%)	411 (39%)
Trisomy 12	28 (14.6%)	47 (14%)	149 (14%)
No abnormalities	47 (24.5%)	57 (18%)	252 (24%)
Deletion 11q23	22 (11.4%)	56(17%)	114 (11%)
Deletion 17p	15 (7.8%)	23 (7%)	122 (12%)
**Karyotype**	N (%)		
Total	139 (100%)		
Complex cytogenetic changes	12 (8.6%)		
Associated FISH abnormalities			
Normal FISH	4 (36%)		
Deletion 17p	4 (36%)		
Trisomy 12	1 (0.9%)		
Deletion 11q23	1 (0.9%)		
Deletion 13q14	1 (0.9%)		
***IGHV* mutation status**	N (%)		
Unmutated	34 (31.2%)		
Mutated	75 (68.8%)		
**Genetic mutations**	N (%)		
*TP53*	8 (8.2% out of 98 patients)		
*MYD88*	7 (11.5% out of 61 patients)		
*NOTCH1*	3 (4.9% out of 61 patients)		
*SF3B1*	4 (6.6% out of 61 patients)		
*FBXW7*	1 (1.6% out of 61 patients)		

Remark: hierarchal model proposed by Dohner et al was used [10]. Those with more than 1 FISH abnormalities would be assigned to a group with worse prognosis.

### Somatic gene mutations and IGHV mutation status

Targeted DNA sequencing showed the most frequently mutated gene to be *MYD88* (11.5%) (794T>C; 85.7%), followed by *TP53* (8.2%) (734G>T; 37.5%), *SF3B1* (6.6%), *NOTCH1* (4.9%) and *FBXW7* (1.6%) (Table 2). No mutation of *XPO1* gene was identified. Unmutated *IGHV* was found in 31.2% of cases (Table 2).

### Correlation between FISH abnormalities, IGHV mutational status, gene mutations and clinical presentation

Patients with del(11)(q23) and del(17p), both unfavourable FISH abnormalities, presented with more advanced-stage disease than patients with del(13)(q14), trisomy 12 or without any FISH abnormalities (p=0.04) (Table 3). The *IGHV* mutation status correlated significantly with disease stage, with more unmutated *IGHV* cases presenting with advanced-stage disease (p<0.01). In addition, patients with high-risk FISH abnormalities, i.e. del(11)(q23) and del(17p), had a significantly more frequent unmutated *IGHV* status than patients with low-risk abnormalities (del(13)(q14), trisomy 12 or no FISH abnormalities) (p <0.01) (Table 4).

**Table 3:** Correlation between FISH abnormalities and stage

	Binet stage			
	AN (%)	BN (%)	CN (%)	
**FISH abnormalities**				
Deletion13q14/trisomy12/normal	93 (60%)	33(21%)	29 (19%)	*p=0.04
Deletion11q23/deletion17p	15 (41%)	12 (32%)	10 (27%)	
	**Binet stage**			
	**AN (%)**	**BN (%)**	**CN (%)**	
***IGHV* mutation**				
Unmutated (Germline)	6 (18%)	18 (53%)	10 (29%)	p<0.01
Mutated	53 (71%)	12 (16%)	10 (13%)	
	***IGHV* mutation**			
	Mutated	Unmutated		
**FISH abnormalities**				
Deletion13q14/trisomy12/normal	59 (77%)	19(23%)		p<0.01
Deletion11q23/deletion17p	6 (29%)	13 (71%)		

**Table 4:** Correlation of *IGHV* mutation status with stage and FISH abnormalities

	Binet stage			
	A N (%)	B N (%)	C N (%)	
***IGHV* mutation**				
Unmutated (Germline)	6 (18%)	18 (53%)	10 (29%)	p<0.01
Mutated	53 (71%)	12 (16%)	10 (13%)	
	***IGHV* mutation**			
	Mutated	Unmutated		
**FISH abnormalities**				
Deletion13q14/trisomy12/normal	59 (77%)	19(23%)		p<0.01
Deletion11q23/deletion17p	6 (29%)	13 (71%)		

Genetic mutations were also significantly associated with FISH abnormalities and *IGHV* mutational status. *TP53* gene mutations were associated with the presence of del(17p) (p<0.01), whereas *SF3B1* gene mutations were associated with normal FISH or del(11q) (p=0.04). *NOTCH1* mutations showed a significant correlation with trisomy 12 (p=0.02). Moreover, the presence of *TP53* mutations was associated with unmutated *IGHV* status (p=0.03) while *MYD88* mutants were associated significantly with mutated *IGHV* (p=0.04) (Table 5).

**Table 5:** Correlation with genetic mutations, FISH abnormalities and IGHV mutational status

	**FISH abnormalities**		**p value**
	**Del(17p)N (%)**	**Non-Del(17p)N (%)**	
Mutated *TP53*	5 (83%)	1 (17%)	p<0.01
Wild-type *TP53*	2 (2.4%)	82 (97.6%)	
	Del(11q) ornormal karyotype by FISHN (%)	Others FISH abnormalities(trisomy 12, Del(13q), Del (17p))N (%)	p=0.04
Mutated *SF3B1*	3 (75%)	1 (25%)	
Wild-type *SF3B1*	13 (26.5%)	36 (73.5%)	
	Trisomy 12N (%)	Non-trisomy 12N (%)	p=0.02
Mutated *NOTCH1*	2 (67%)	1 (33%)	
Wild-type *NOTCH1*	7 (14%)	43 (86%)	
	**IGHV mutational status**		p value
	Mutated *IGHV*N (%)	Unmutated *IGHV*N (%)	
Mutated *TP53*	2 (28.6%)	5 (71.4%)	
Wild-type *TP53*	62 (70%)	27 (30%)	p=0.03
Mutated *MYD88*	7 (100%)	0 (0%)	
Wild-type *MYD88*	20 (38.5%)	32 (61.5%)	p=0.04

### Correlation between FISH abnormalities, IGHV mutational status, gene mutations and survival

**Figure 1:**
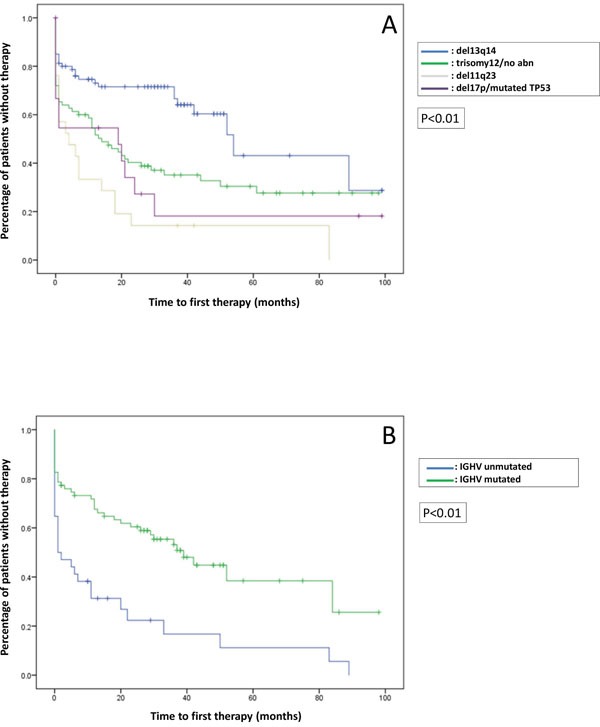
Correlation between time to first therapy (TTFT) and genetic abnormalities **(A)** TTFT stratified according to the FISH abnormlities. **(B)** TTFT stratified according to the *IGHV* mutation status.

**Figure 2:**
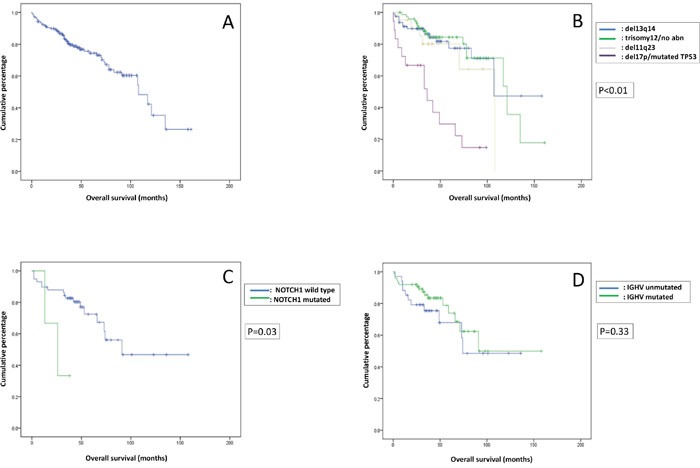
Overall survival (OS) of all patients **(A)** OS of the whole cohort of CLL patients. **(B)** OS stratified according to the FISH abnormalities. **(C)** OS stratified according to the *NOTCH1* mutations. **(D)** OS stratefied according to the *IGHV* mutation status.

The median TTFT was 21 (0-99) months. A significantly longer TTFT was found in patients having del(13)(q14) compared with patients having trisomy12/no abnormalities, del(11)(q23) or del(17p)/*TP53* mutations (p<0.01) (Figure 1A), and in patients having mutated *IGHV* compared with patients having unmutated *IGHV* (p<0.01) (Figure 1B). Mutations of *NOTCH1*, *SF3B1*, *MYD88* and *FBXW7* did not impact on TTFT. With a median follow up of 39 months, the median OS of the whole cohort was 108 (0–161) months (Figure 2A). Patients with del(17p)/*TP53* mutations had the worst OS, compared with those with other FISH abnormalities (p<0.01) (Figure 2B). The presence of *NOTCH1* mutations was also associated with a significantly worse OS (p=0.03) (Figure 2C). Patients with mutated *IGHV* had longer median OS than those with unmutated *IGHV* (106 versus 87 months), but the difference was not statistically significant (p=0.33) (Figure 2D).

### Outcome of patients who received treatment

**Figure 3:**
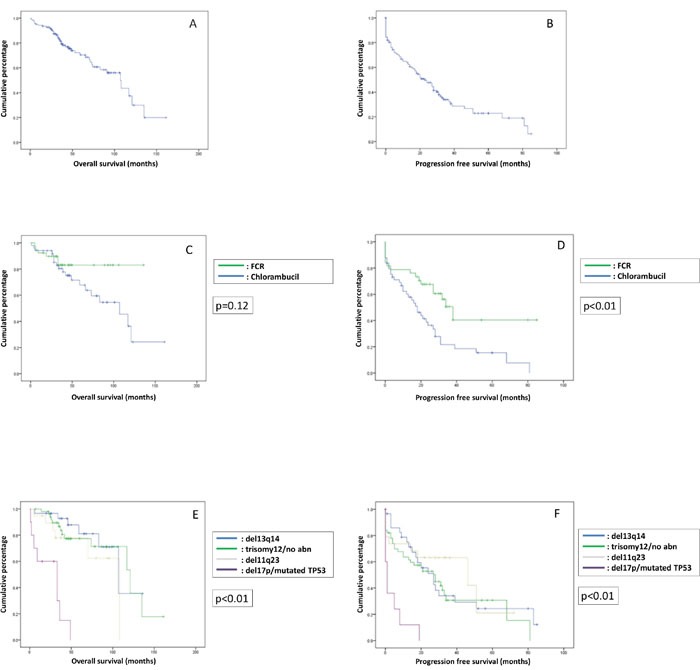
Overall survival (OS) and progression free survival (PFS) of patients who received treatment **(A)** OS of the treated patients. **(B)** PFS of the treated patients. **(C)** OS of the treated patients stratified according to the therapies received (FCR *vs* chlorambucil). **(D)** PFS of the treated patients stratified according to the therapies received (FCR *vs* chlorambucil). **(E)** OS of the treated patients stratified according to the FISH abnormalities. **(F)** PFS of the treated patients stratified according to the FISH abnormalities.

One hundred and twenty-three patients required treatment (Table 6). With a median follow-up of 44 (1-161) months, the median OS and PFS were 107 (1-161) months (Figure 3A) and 23 (0-85) months (Figure 3B). In patients treated with chlorambucil (N=51), the median OS and PFS were 107 (1-161) months and 17 (0-81) months (Figure 3C and 3D), respectively. However, for patients treated with FCR (N=39), the median OS was not reached and the median PFS was 38 (0-85) months (Figure 3C and 3D). Compared with chlorambucil-treated patients, FCR-treated patients were younger, more likely to have advanced-stage disease and unmutated *IGHV* (p<0.01) (Table 7). Del(17p) (N=10) was associated with a significantly inferior survival (p<0.01), compared with other FISH abnormalities, with patients having a median OS of 33 months and a PFS of only 1 month (Figure 3E and 3F).

**Table 6:** Summary of chemotherapy regimens of 123 patients

Chemotherapy regimen used	N (%)
**Alkylator based**	
Chlorambucil alone	51 (41.5%)
**Purine analogue based**	
Fludarabine (without rituximab)	11 (8.9%)
**Chemoimmunotherapy**	
Rituximab and fludarabine (excluding FCR)	3 (2.4%)
FCR	39 (31.7%)
RB	5 (4.1%)
**Others**	14 (11.4%)

FCR: fludarabine, cyclophosphamide and rituximab, RB: rituximab and bendamustine, Others include CHOP: cyclophosphamide, doxorubicin, vincristine and prednisolone, CVP: cyclophosphamide, vincristine and prednisolone, R-CHOP: rituximab plus CHOP, R-CVP: rituximab plus CVP, rituximab alone, dexamethasone alone.

**Table 7:** Correlation between treatment groups and clinical characteristics, FISH abnormalities and *IGHV* mutation status

	Chlorambucil	FCR	p value
Median age (years) (range)	68 (44-91)	56 (26-76)	p<0.01
Binet stage	N (%)		
A	27 (53%)	5 (12.8%)	
B	14 (27.4%)	18 (46.2%)	p<0.01
C	10 (19.6%)	16 (41%)	
Total	51	39	
*IGHV* mutational status	N (%)		
Unmutated	7 (21%)	18 (72%)	
Mutated	26 (79%)	7 (28%)	p<0.01
Total	33	25	
FISH abnormalities	N (%)		
Del13q14/normal/trisomy12	34 (79%)	24 (63%)	
Del11q23/Del17p	9 (21%)	14 (37%)	p=0.1
Total	43	38	

### Richter's transformation

Clinical information on Richter's transformation was available in 139 patients. With a median follow-up period of 36 months, 5 patients developed Richter's transformation. FISH abnormalities, *IGHV* mutation status, somatic gene mutations, or the types of therapeutic agent used were not correlated with the development of Richter's transformation.

## DISCUSSION

Our study reports on one of the largest series of Chinese CLL patients. There are several important observations. Firstly, the median age at diagnosis of our patients is 64, which is much lower than the median age of 72 reported for Caucasian CLL patients [22]. Interestingly, our result is also comparable with the median age at diagnosis reported in other studies of CLL in Asian populations, which ranges from 57 to 66 [23–26]. Furthermore, the lower median age at diagnosis is also observed in Chinese residents in the Western world, suggesting that there may be a genetic basis for CLL to present at a younger age in Chinese population [4]. Nonetheless, the preferential referral of younger CLL patients to the participating specialist centres in this study may also partly contribute to the observed difference.

On the other hand, the prevalence and pattern of cytogenetic abnormalities in Chinese patients were similar to those of Caucasian patients. This observation was similarly reported in previous series of Chinese CLL patients [24, 27] (Table 8). Isolated del(13q)(q14) was the most prevalent cytogenetic abnormality, occurring in 41.7% of our cases. This frequency is similar to that of 36% in the original study that documented a favorable prognostic impact of this aberration [10]. In a recent study from the CLL research consortium [28] revisiting the hierarchal prognostic model in the era of chemoimmunotherapy, isolated 13q14 deletion occurred with a similar frequency of 39%, and was also associated with a favorable outcome.

**Table 8:** Comparison of FISH abnormalities in Chinese patients with CLL

	Wu *et al*. (N=83^#^) [27]	Current study (N=192)
13q14 deletion	38 (45.8%)	80 (41.7%)
Trisomy 12	17 (20.5%)	28(14.6%)
No abnormalities on FISH	25 (30.1%)	47 (24.5%)
11q23 deletion	11 (13.3%)	22(11.4%)
17p deletion	9 (10.8%)	15 (7.8%)

# patients with more than one abnormality were counted more than once.

Mutations of *NOTCH1*, *SF3B1*, and *FBXW7* in our cohort were again similar in frequencies compared with CLL patients from Western countries (Table 9). In contrast, mutations of *MYD88* were more frequently identified in Chinese (10.8%) than Caucasians patients [29–31]. Interestingly, a similarly higher frequency of *MYD88* mutations in Chinese CLL patients was also observed in another study [25]. MYD88 is an important protein involved in the toll-like receptor complex. Mutations in *MYD88* lead to constitutive activation of NF-*k*B [30]. It also increases the release of interleukin-6 and chemokine ligands 2, 3 and 4, resulting in recruitment of macrophages and T lymphocytes [32]. All these changes create a favourable microenvironmental niche for the survival of CLL cells. *MYD88* mutations have also been implicated as an early event in the pathogenesis of CLL [33], and associated with mutated *IGHV* and better overall survival [34]. The rate of *TP53* mutations was slightly higher than expected compared with Caucasian CLL, considering the lower prevalence of *IGHV* unmutated CLL among Chinese patients [35]. The overall prognostic impact of *MYD88* and *TP53* mutations in Chinese CLL patients, however, requires further evaluation in future studies.

**Table 9:** Prevalence of novel genetic mutations

	Caucasians			Chinese	
	Jeromin et al [29]	Puente et al[30]	Cortese et al [31]	Xia et al[25]	Current
TP53 (%)	7.1	-	5.2	8	8.2
MYD88 (%)	1.5	2.9	2.3	10	11.5
NOTCH1 (%)	12.3	12.2	4.7	3	4.9
SF3B1 (%)	9.0	-	3.6	4	6.6
FBXW7 (%)	2.5	-	-	-	1.6

Remark: P<0.01 comparing patient cohort from Jeromin et al and the current study. Chi square was not performed for comparison with other groups as some of the mutations were not reported.

The correlations among genetic mutations, *IGHV* mutational status and FISH abnormalities reported in Caucasians [17, 29] were also similarly seen in our cohort. In particular, *NOTCH1* mutations were associated with trisomy 12. In Caucasian patients, *NOTCH1* mutations were enriched from an overall frequency of 10% to that of 24% in patients having trisomy 12. The presence of *NOTCH1* mutations in trisomy 12 also confers an inferior survival [19]. The prognostic effect of *NOTCH1* mutations was also observed in our Chinese patients.

The prevalence of mutated *IGHV* in the Western population was reported to be around 55-64% [13, 29, 36]. Early reports have demonstrated a high prevalence of mutated *IGHV* in Chinese patients [37], and this was confirmed in a larger cohort of Chinese patients [35]. In the current study, 68% of our patients had mutated *IGHV*, slightly higher than that in the Western populations. Further studies are required to confirm if this higher prevalence of *IGHV* may be associated with a better outcome in Chinese patients with CLL.

Cytogenetic abnormalities significantly impact on TTFT. In our study, patients with del(13)(q14) had a significantly longer TTFT compared with the rest of the cohort. However, the difference among the remaining three cytogenetic groups was not apparent. Van Dyke et al [28] have shown similar findings, with the TTFT from diagnosis being 35 months, 30 months, 22 months and 22 months for patients with normal FISH, trisomy 12, del(11)(q23) and del(17p), respectively. These findings illustrate that CLL with del(17p) may initially run an indolent course, and treatment should not be commenced based only on cytogenetic findings. In contrast, CLL with del(17p) was associated with a significantly worse survival outcome, reflecting its poor response to current chemoimmunotherapy once treatment is needed.

We also showed that patients with mutated *IGHV* had a longer TTFT. This is in accordance with results reported in Western populations [38, 39]. A superior, though not statistically significant, OS was also seen in patients with mutated *IGHV*, indicating that the prognostic relevance of *IGHV* mutation was also observed in Chinese patients.

However, there were some inherent limitations of the study. Firstly, the percentage of our patients presenting with Binet stage A (56%) was lower than that reported in the Western World [40]. Routine health-check including blood tests is not commonly practiced in the region especially during the early recruitment period of the study, and this may lead to the under-diagnosis of asymptomatic Binet stage A patients. In addition, the heterogeneity in treatments would affect the outcome of patients in this study. In Hong Kong and Singapore, healthcare system is mostly government subsidized, and accessibility to expensive medications is dependent on reimbursement policies. Chlorambucil was thus the mainstay of treatment, especially in early study period. As a result, a large proportion of patients with Binet stage A were given chlorambucil. Furthermore, the lack of standardized criteria for treatment initiation and the imbalance in baseline characteristics of the two treatment groups (FCR *vs* chlorambucil) probably had introduced bias in our analysis. In the FCR group, there was a higher proportion of patients with unmutated IGHV, as compared with the chlorambucil. This might explain the lack of statistical significant difference in OS between IGHV mutated and unmutated patient groups.

In conclusion, in a population where the incidence of CLL is only about 10% of that in Western countries, we have shown in one of the largest cohorts of Chinese patients studied at diagnosis that FISH and genetic abnormalities are similar to those previously reported in Western patients. *MYD88* mutations and mutated *IGHV* were apparently more frequent. The prognostic significance of hierarchal FISH abnormalities, genetic mutations and *IGHV* mutation status in refining the prognostic stratification is applicable to Chinese patients.

## MATERIALS AND METHODS

### Patients

CLL patients of Chinese ethnicity diagnosed and treated in participating haematology centres from Hong Kong and Singapore were analysed. Records of ethnicities of the patients were retrieved from the case notes. Patients of other ethnic groups were excluded from the study. Diagnosis of CLL was based on standard morphological and immunophenotypical criteria. Indications for treatment followed those of the iwCLL 2008 guidelines [9] and the initiation of treatment was at the discretion of the treating physicians.

There were two groups of patients from the Hong Kong cohort. The first was a retrospective group identified between January 2003 to June 2010. The second group was a prospective group collected between July 2010 to June 2013. Patients from Singapore were from a retrospective group identified from January 1999 to July 2015. All samples were taken from treatment-naïve patient with newly diagnosed CLL and were collected at the time of diagnosis.

### Cytogenetic and FISH analysis

A 72-hour synchronized culture with stimulation by 12-O-tetradecanoylphorbol-13-acetate (TPA) was set up on marrow or peripheral blood lymphocytes for conventional cytogenetic study. Metaphase chromosomes were banded by trypsin/Giemsa and karyotyped according to the International System for Human Cytogenetic Nomenclature, ISCN (2013). Cell pellets in Carnoy's fixative that were processed for cytogenetics study were used for FISH analysis. Slides were prepared and aged overnight at 65°C. Two separate hybridizations using probe mix from Vysis (Abbott Molecular Inc, IL) were performed. Mix 1 included the SpectrumGreen-labeled *ATM* (11q22.3) and SpectrumOrange-labeled *TP53* (17p13.1) probes. Mix 2 included the SpectrumGreen-labeled D12Z3 (CEP12), SpectrumOrange-labeled D13S319 (13q14.3) and SpectrumAqua-labeled LAMP1 (13q34) probes. The sample and probes were co-denatured at 75°C for 5 minutes and hybridized overnight at 37°C. Slides were washed in 0.4 × SSC/0.3% NP40, pH7.0, at 70°C for 2 minutes, and then in 2 × SSC/0.1% NP40, pH7.0 at room temperature for 1 minute to remove non-specific binding. Slides were counterstained with 4′,6-diamidino-2-phenylindole (DAPI II, 125 ng/ml, Vysis). Both metaphases and interphases were analyzed. Depending on the mitotic index, 2 – 30 metaphases were analyzed for each case at each probe locus. A total of 200 interphase cells at 1000x magnification were scored with single band-pass filters independently by two observers using a Nikon (model 80i) fluorescence microscope. The Isis v5.4.8 platform (MetaSystems, Altlussheim, Germany) was used for image capture and processing. The cut-off values for a 200-cell score were: (A) trisomy 12, 1.8%, (B) del(11)(q22.3), heterozygous 5.7% and homozygous 1.4%, (C) del(13)(q14.3), heterozygous 4.8% and homozygous 1.8% and (D) del(17)(p13.1), heterozygous 6.2% and homozygous 1.8%. FISH results were reported according to ISCN (2013).

### *IGHV* mutation status

The analysis of *IGHVDJ* genes was carried out on peripheral blood leukaemic cells. Polymerase chain reaction (PCR) amplification and sequence analysis of *IGHVDJ* rearrangements were performed on either genomic DNA (gDNA) or cDNA using sense family specific VH primers (framework region 1 [FR1] or VH leader primers), combined with consensus JH primers as previously described [41]. PCR products were sequenced directly or after a cloning procedure, using 3130 Genetic Analyzer (Life Technologies, Carlsbad, CA). Productive rearrangements were analyzed by the IMGT database (http://www.imgt.org, Montepellier, France) [42] and the IMGT/V-QUEST tool (version 3.3.0) [43]. Percentage of identity to the closest germline *IGHV* allele was evaluated [41].

### Somatic gene mutation analysis

Targeted sequencing of *TP53* (exons 4-11), *NOTCH1* (exons 33-34), *SF3B1* (exons 10-16), *FBXW7* (exons 8-12), *MYD88* (exons 4-5) and *XPO1* (exons 14-15) was performed with the Genome Sequencer Junior instrument (Roche-454) (Roche, Mannheim, Germany). Primers sequences for all amplicons are available upon request. Each sequencing run analyzed 93 amplicons, consisting of the target genes of 3 individual patients, and a ∼1000-fold coverage for all amplicons was achieved.

Genomic DNA was amplified using FastStart High Fidelity Polymerase (Roche, Mannheim, Germany) and oligonucleotides containing the exon-specific sequences, along with 10-bp multiplex identifiers (MID) tags for multiplexing and amplicon library A and B sequencing adapters. Each PCR product was purified using Agentcourt AMPure XP beads (Beckman Coulter, Brea, CA) and quantified by the Quant-iT PicoGreen dsDNA kit (Life Technologies, Eugene, Oregon). All amplicons were pooled together at equimolar ratios, added to emulsion PCR at a ratio of 0.8 molecules per bead and subjected to Roche-454 pyrosequencing. The analysis was performed using Amplicon Variant Analysis software (AVA, Roche). After the exclusion of synonymous variants and polymorphisms, all the identified mutations were confirmed with Sanger DNA sequencing. Additionally, *TP53* mutations were investigated by conventional Sanger DNA sequencing with an ABI PRISM 3100 Genetic Analyzer (Applied Biosystems, Foster City, CA). Sequences were compared with the corresponding germline RefSeq sequences with the Mutation Surveyor Version 2.41 software package (SoftGenetics) after both automated and manual curation. PCR conditions were applied as previously described [44].

### Treatment

Therapeutic regimens evolved during the study period and were heterogeneous. They included alkylator-based (chlorambucil) and purine analogue-based (fludarabine) regimens, and chemoimmunotherapy (fludarabine, cyclophosphamide and rituximab, FCR). In general, patients <65 years old and medically fit received intensive chemoimmunotherapy. Patients ≥65 years old or with more than one comorbidities were treated less intensively. All patients consented to blood sample collection and treatment.

### Statistical analysis

Time to first treatment (TTFT) was measured from diagnosis to initiation of first treatment. Overall survival (OS) was measured from diagnosis to death or last follow-up. Progression-free survival (PFS) was measured from treatment initiation to disease progression, death or last follow-up. Data were censored at June 2015. Risks based on FISH results were stratified according to the algorithm proposed by Dohner et al.[10] Categorical variables were analysed using Chi-square test. Survival analysis was performed with the Kaplan-Meier method and log-rank test. P values were 2-sided and considered significant when <0.05. Statistical analysis was performed using the SPSS software (version 21.0, IBM Corporation, Armonk, NY).

### Ethics approval

The project is approved by the Institutional Review Board of the University of Hong Kong/Hospital Authority Hong Kong West Cluster (IRB ref number: UW 12-304) and Singhealth Centralized Institutional Review Board of Singapore (CIRB ref number: 2012/375/F).

## References

[1] Swerdlow SH, International Agency for Research on Cancer. and World Health Organization (2008). WHO classification of tumours of haematopoietic and lymphoid tissues.

[2] Howlader N NA, Krapcho M, Garshell J, Miller D, Altekruse SF, Kosary CL, Yu M, Ruhl J, Tatalovich Z, Mariotto A, Lewis DR, Chen HS, Feuer EJ, http://seer.cancer.gov/csr/1975_2012/, based on November 2014 SEER data submission, posted to the SEER web site, April 2015. (National Cancer Institute. Bethesda, MD). SEER Cancer Statistics Review, 1975-2012, National Cancer Institute.

[3] Chihara D, Ito H, Matsuda T, Shibata A, Katsumi A, Nakamura S, Tomotaka S, Morton LM, Weisenburger DD, Matsuo K (2014). Differences in incidence and trends of haematological malignancies in Japan and the United States. British journal of haematology.

[4] Mak V, Ip D, Mang O, Dalal C, Huang S, Gerrie A, Gillan T, Ramadan KM, Toze C, Au WY (2014). Preservation of lower incidence of chronic lymphocytic leukemia in Chinese residents in British Columbia: a 26-year survey from 1983 to 2008. Leukemia & lymphoma.

[5] Gale RP, Cozen W, Goodman MT, Wang FF, Bernstein L (2000). Decreased chronic lymphocytic leukemia incidence in Asians in. Los Angeles County. Leuk Res.

[6] Yang SM, Li JY, Gale RP, Huang XJ (2015). The mystery of chronic lymphocytic leukemia (CLL): Why is it absent in Asians and what does this tell us about etiology, pathogenesis and biology?. Blood Rev.

[7] Rai KR, Sawitsky A, Cronkite EP, Chanana AD, Levy RN, Pasternack BS (1975). Clinical staging of chronic lymphocytic leukemia. Blood.

[8] Binet JL, Lepoprier M, Dighiero G, Charron D, D’Athis P, Vaugier G, Beral HM, Natali JC, Raphael M, Nizet B, Follezou JY (1977). A clinical staging system for chronic lymphocytic leukemia: prognostic significance. Cancer.

[9] Hallek M, Cheson BD, Catovsky D, Caligaris-Cappio F, Dighiero G, Dohner H, Hillmen P, Keating MJ, Montserrat E, Rai KR, Kipps TJ (2008). International Workshop on Chronic Lymphocytic L. Guidelines for the diagnosis and treatment of chronic lymphocytic leukemia: a report from the International Workshop on Chronic Lymphocytic Leukemia updating the National Cancer Institute-Working Group 1996 guidelines. Blood.

[10] Dohner H, Stilgenbauer S, Benner A, Leupolt E, Krober A, Bullinger L, Dohner K, Bentz M, Lichter P (2000). Genomic aberrations and survival in chronic lymphocytic leukemia. N Engl J Med.

[11] Hallek M, Fischer K, Fingerle-Rowson G, Fink AM, Busch R, Mayer J, Hensel M, Hopfinger G, Hess G, von Grunhagen U, Bergmann M, Catalano J, Zinzani PL (2010). Addition of rituximab to fludarabine and cyclophosphamide in patients with chronic lymphocytic leukaemia: a randomised, open-label, phase 3 trial. Lancet.

[12] Damle RN, Wasil T, Fais F, Ghiotto F, Valetto A, Allen SL, Buchbinder A, Budman D, Dittmar K, Kolitz J, Lichtman SM, Schulman P, Vinciguerra VP (1999). Ig V gene mutation status and CD38 expression as novel prognostic indicators in chronic lymphocytic leukemia. Blood.

[13] Hamblin TJ, Davis Z, Gardiner A, Oscier DG, Stevenson FK (1999). Unmutated Ig V(H) genes are associated with a more aggressive form of chronic lymphocytic leukemia. Blood.

[14] Oscier DG, Rose-Zerilli MJ, Winkelmann N, Gonzalez de Castro D, Gomez B, Forster J, Parker H, Parker A, Gardiner A, Collins A, Else M, Cross NC (2013). The clinical significance of NOTCH1 and SF3B1 mutations in the UK LRF CLL4 trial. Blood.

[15] Stilgenbauer S, Schnaiter A, Paschka P, Zenz T, Rossi M, Dohner K, Buhler A, Bottcher S, Ritgen M, Kneba M, Winkler D, Tausch E, Hoth P (2014). Gene mutations and treatment outcome in chronic lymphocytic leukemia: results from the CLL8 trial. Blood.

[16] Schnaiter A, Paschka P, Rossi M, Zenz T, Buhler A, Winkler D, Cazzola M, Dohner K, Edelmann J, Mertens D, Kless S, Mack S, Busch R (2013). NOTCH1, SF3B1, and TP53 mutations in fludarabine-refractory CLL patients treated with alemtuzumab: results from the CLL2H trial of the GCLLSG. Blood.

[17] Chiaretti S, Marinelli M, Del Giudice I, Bonina S, Piciocchi A, Messina M, Vignetti M, Rossi D, Di Maio V, Mauro FR, Guarini A, Gaidano G, Foa R (2014). NOTCH1, SF3B1, BIRC3 and TP53 mutations in patients with chronic lymphocytic leukemia undergoing first-line treatment: correlation with biological parameters and response to treatment. Leuk Lymphoma.

[18] Rossi D, Fangazio M, Rasi S, Vaisitti T, Monti S, Cresta S, Chiaretti S, Del Giudice I, Fabbri G, Bruscaggin A, Spina V, Deambrogi C, Marinelli M (2012). Disruption of BIRC3 associates with fludarabine chemorefractoriness in TP53 wild-type chronic lymphocytic leukemia. Blood.

[19] Del Giudice I, Rossi D, Chiaretti S, Marinelli M, Tavolaro S, Gabrielli S, Laurenti L, Marasca R, Rasi S, Fangazio M, Guarini A, Gaidano G, Foa R (2012). NOTCH1 mutations in +12 chronic lymphocytic leukemia (CLL) confer an unfavorable prognosis, induce a distinctive transcriptional profiling and refine the intermediate prognosis of +12 CLL. Haematologica.

[20] Rossi D, Bruscaggin A, Spina V, Rasi S, Khiabanian H, Messina M, Fangazio M, Vaisitti T, Monti S, Chiaretti S, Guarini A, Del Giudice I, Cerri M (2011). Mutations of the SF3B1 splicing factor in chronic lymphocytic leukemia: association with progression and fludarabine-refractoriness. Blood.

[21] Foa R, Del Giudice I, Guarini A, Rossi D, Gaidano G (2013). Clinical implications of the molecular genetics of chronic lymphocytic leukemia. Haematologica.

[22] Eichhorst B, Robak T, Montserrat E, Ghia P, Hillmen P, Hallek M, Buske C (2015). Chronic lymphocytic leukaemia: ESMO Clinical Practice Guidelines for diagnosis, treatment and follow-up. Ann Oncol.

[23] Gunawardana C, Austen B, Powell JE, Fegan C, Wandroo F, Jacobs A, Pratt G, Moss P (2008). South Asian chronic lymphocytic leukaemia patients have more rapid disease progression in comparison to White patients. British journal of haematology.

[24] Qiu HX, Xu W, Cao XS, Zhou M, Shen YF, Xu YL, Sun XM, Liu Q, Wang R, Qiu HR, Wang JS, Li JY (2008). Cytogenetic characterisation in Chinese patients with chronic lymphocytic leukemia: a prospective, multicenter study on 143 cases analysed with interphase fluorescence in situ hybridisation. Leukemia & lymphoma.

[25] Xia Y, Fan L, Wang L, Gale RP, Wang M, Tian T, Wu W, Yu L, Chen YY, Xu W, Li JY (2015). Frequencies of SF3B1, NOTCH1, MYD88, BIRC3 and IGHV mutations and TP53 disruptions in Chinese with chronic lymphocytic leukemia: disparities with Europeans. Oncotarget.

[26] Tomomatsu J, Isobe Y, Oshimi K, Tabe Y, Ishii K, Noguchi M, Hirano T, Komatsu N, Sugimoto K (2010). Chronic lymphocytic leukemia in a Japanese population: varied immunophenotypic profile, distinctive usage of frequently mutated IGH gene, and indolent clinical behavior. Leukemia & lymphoma.

[27] Wu SJ, Lin CT, Huang SY, Lee FY, Liu MC, Hou HA, Chen CY, Ko BS, Chou WC, Yao M, Tang JL, Tsay W, Tien HF (2013). Chromosomal abnormalities by conventional cytogenetics and interphase fluorescence in situ hybridization in chronic lymphocytic leukemia in Taiwan, an area with low incidence--clinical implication and comparison between the West and the East. Ann Hematol.

[28] Van Dyke DL, Werner L, Rassenti LZ, Neuberg D, Ghia E, Heerema NA, Dal Cin P, Dell Aquila M, Sreekantaiah C, Greaves AW, Kipps T, Kay NE (2016). The Dohner fluorescence in situ hybridization prognostic classification of chronic lymphocytic leukaemia (CLL): the CLL Research Consortium experience. British journal of haematology.

[29] Jeromin S, Weissmann S, Haferlach C, Dicker F, Bayer K, Grossmann V, Alpermann T, Roller A, Kohlmann A, Haferlach T, Kern W, Schnittger S (2014). SF3B1 mutations correlated to cytogenetics and mutations in NOTCH1, FBXW7, MYD88, XPO1 and TP53 in 1160 untreated CLL patients. Leukemia.

[30] Puente XS, Pinyol M, Quesada V, Conde L, Ordonez GR, Villamor N, Escaramis G, Jares P, Bea S, Gonzalez-Diaz M, Bassaganyas L, Baumann T, Juan M (2011). Whole-genome sequencing identifies recurrent mutations in chronic lymphocytic leukaemia. Nature.

[31] Cortese D, Sutton LA, Cahill N, Smedby KE, Geisler C, Gunnarsson R, Juliusson G, Mansouri L, Rosenquist R (2014). On the way towards a ’CLL prognostic index’: focus on TP53, BIRC3, SF3B1, NOTCH1 and MYD88 in a population-based cohort. Leukemia.

[32] Burger JA, Quiroga MP, Hartmann E, Burkle A, Wierda WG, Keating MJ, Rosenwald A (2009). High-level expression of the T-cell chemokines CCL3 and CCL4 by chronic lymphocytic leukemia B cells in nurselike cell cocultures and after BCR stimulation. Blood.

[33] Landau DA, Carter SL, Stojanov P, McKenna A, Stevenson K, Lawrence MS, Sougnez C, Stewart C, Sivachenko A, Wang L, Wan Y, Zhang W, Shukla SA (2013). Evolution and impact of subclonal mutations in chronic lymphocytic leukemia. Cell.

[34] Martinez-Trillos A, Pinyol M, Navarro A, Aymerich M, Jares P, Juan M, Rozman M, Colomer D, Delgado J, Gine E, Gonzalez-Diaz M, Hernandez-Rivas JM, Colado E (2014). Mutations in TLR/MYD88 pathway identify a subset of young chronic lymphocytic leukemia patients with favorable outcome. Blood.

[35] Marinelli M, Ilari C, Xia Y, Del Giudice I, Cafforio L, Della Starza I, Raponi S, Mariglia P, Bonina S, Yu Z, Yang W, Qiu L, Chan T (2016). Immunoglobulin gene rearrangements in Chinese and Italian patients with chronic lymphocytic leukemia. Oncotarget.

[36] Del Giudice I, Mauro FR, De Propris MS, Santangelo S, Marinelli M, Peragine N, Di Maio V, Nanni M, Barzotti R, Mancini F, Armiento D, Paoloni F, Guarini A (2011). White blood cell count at diagnosis and immunoglobulin variable region gene mutations are independent predictors of treatment-free survival in young patients with stage A chronic lymphocytic leukemia. Haematologica.

[37] Chen L, Zhang Y, Zheng W, Wu Y, Qiao C, Fan L, Xu W, Li J (2008). Distinctive IgVH gene segments usage and mutation status in Chinese patients with chronic lymphocytic leukemia. Leuk Res.

[38] Wierda WG, O’Brien S, Wang X, Faderl S, Ferrajoli A, Do KA, Garcia-Manero G, Cortes J, Thomas D, Koller CA, Burger JA, Lerner S, Schlette E (2011). Multivariable model for time to first treatment in patients with chronic lymphocytic leukemia. Journal of clinical oncology.

[39] Morilla A, Gonzalez de Castro D, Del Giudice I, Osuji N, Else M, Morilla R, Brito Babapulle V, Rudenko H, Matutes E, Dearden C, Catovsky D, Morgan GJ (2008). Combinations of ZAP-70, CD38 and IGHV mutational status as predictors of time to first treatment in CLL. Leukemia & lymphoma.

[40] Abrisqueta P, Pereira A, Rozman C, Aymerich M, Gine E, Moreno C, Muntanola A, Rozman M, Villamor N, Hodgson K, Campo E, Bosch F, Montserrat E (2009). Improving survival in patients with chronic lymphocytic leukemia (1980-2008): the Hospital Clinic of Barcelona experience. Blood.

[41] Ghia P, Stamatopoulos K, Belessi C, Moreno C, Stilgenbauer S, Stevenson F, Davi F, Rosenquist R (2007). European Research Initiative on CLL. ERIC recommendations on IGHV gene mutational status analysis in chronic lymphocytic leukemia. Leukemia.

[42] Lefranc MP, Giudicelli V, Ginestoux C, Jabado-Michaloud J, Folch G, Bellahcene F, Wu Y, Gemrot E, Brochet X, Lane J, Regnier L, Ehrenmann F, Lefranc G, Duroux P. IMGT (2009). the international ImMunoGeneTics information system. Nucleic Acids Res.

[43] Brochet X, Lefranc MP, Giudicelli V (2008). IMGT/V-QUEST: the highly customized and integrated system for IG and TR standardized V-J and V-D-J sequence analysis. Nucleic Acids Res.

[44] Pospisilova S, Gonzalez D, Malcikova J, Trbusek M, Rossi D, Kater AP, Cymbalista F, Eichhorst B, Hallek M, Dohner H, Hillmen P, van Oers M, Gribben J (2012). ERIC recommendations on TP53 mutation analysis in chronic lymphocytic leukemia. Leukemia.

